# Autonomy, Competence and Non-interference

**DOI:** 10.1007/s10730-017-9344-1

**Published:** 2017-12-30

**Authors:** Joseph T. F. Roberts

**Affiliations:** Politics, School of Social Sciences, University of Manchester, Arthur Lewis Building, Oxford Road, Manchester, M13 9PL UK

**Keywords:** Autonomy, Paternalism, Competence, Liberalism, Non-interference

## Abstract

In light of the variety of uses of the term autonomy in recent bioethics literature, in this paper, I suggest that competence, not being as contested, is better placed to play the anti-paternalistic role currently assigned to autonomy. The demonstration of competence, I will argue, can provide individuals with robust spheres of non-interference in which they can pursue their lives in accordance with their own values. This protection from paternalism is achieved by granting individuals rights to non-interference upon demonstration of competence. In this paper, I present a risk-sensitive account of competence as a means of grounding rights to non-interference. On a risk-sensitive account of competence individuals demonstrate their competence by exercising three capacities to the extent necessary to meet a threshold determined by the riskiness of the decision. These three capacities are the capacity to (i) acquire knowledge, (ii) use instrumental rationality, and (iii) form and revise a life plan.

## Introduction

In this paper, I argue that, in light of the mismatch between the competing interpretations of autonomy in the wider philosophical literature and the interpretation given to the term autonomy in a liberal bioethics (which encompasses only some of the uses in the wider literature), we should consider moving towards talk of “competence” rather than “autonomy” when allocating decision-making authority to people in the clinical context. In a liberal bioethics, the value of autonomy is taken to require that medical practitioners be sensitive to the individual’s values and desires regarding healthcare interventions. Autonomy, hence, plays an anti-paternalistic role by allowing individuals to live their own lives in accordance with their own values. In the wider philosophical literature on autonomy, however, it encompasses much more than that. Autonomy, for example, is normally taken also to require the lack of coercion, deception or manipulation (Noggle 2005, 87). Some authors take the fact that autonomy is incompatible with coercion, deception and manipulation to imply that autonomy requires independence of some sort (Dworkin 1988). Autonomy is also often taken to require authenticity; that is, that an individual’s values be their *own* in some sense (Hyun 2001; Noggle 2005; Watson 1975). Moreover, autonomy is also closely linked to rationality and knowledge, as ignorance, (Savulescu 2007; Savulescu and Momeyer 1997) and certain forms of irrationality (delusions for example) are often taken to undermine autonomy.

In light of this variety of uses, in this paper, I suggest that competence, not being as contested, is better placed to play the anti-paternalistic role currently assigned to autonomy. The demonstration of competence, I will argue, can provide individuals with robust spheres of non-interference in which they can pursue their lives in accordance with their own values. This protection from paternalism is achieved by granting individuals rights to non-interference upon the demonstration of competence. In this paper, I present a risk-sensitive account of competence as a means of grounding rights to non-interference (Brock 1991; Buchanan and Brock 1986). On a risk-sensitive account of competence an individual demonstrates their competence by exercising three capacities to the extent necessary to meet a threshold determined by the riskiness of the decision. These three capacities are the capacity to (i) acquire knowledge, (ii) use instrumental rationality, and (iii) form and revise a life plan.

Before turning to arguing for replacing talk of autonomy with a risk-sensitive account of competence, it will be useful to outline the structure of the argument that follows. In order to ground the problem, in the following section, I will briefly discuss some of the panoply of meanings currently attributed to autonomy and argue that many of these fail to explain or justify the primary role of autonomy in a liberal bioethics. Secondly, I argue that, in clinical ethics, autonomy primarily plays an anti-paternalistic role by protecting people’s ability to live their own lives in accordance with their own values. In the section entitled “Competence and Decisional Authority”, I develop an account of competence which grounds robust rights to non-interference in order to show that competence can play the anti-paternalistic role currently attributed to autonomy. By grounding rights to non-interference, the demonstration of competence provides people with space in which to organise their own lives in accordance with their own values. With this account of rights against non-interference in hand, in the section entitled “Risk-Sensitive Competence”, I take up the task of establishing how competence determinations should be made, arguing for a risk-sensitive account of competence which yields competence determinations which are compatible with the requirements respect for autonomy is normally taken to impose.

## Autonomy and Its Role in a Liberal Bioethics

In this section, I aim to show two things: firstly, that the term “autonomy” is used in a number of ways in the literature and, secondly, that autonomy’s core role in a liberal bioethics is the protection of people’s ability to live their lives in accordance with their own values. This section, hence, lays the foundation for the argument of the rest of the paper: that competence is better suited to play the anti-paternalistic role currently assigned to autonomy. If talk of autonomy in the philosophical literature in general can be shown to be wider than the role it should play in a liberal bioethics, we have reason to re-focus the discussion surrounding the core problem autonomy is used to address: when should people be entitled to make their own decisions regarding their own healthcare in light of their own values.

Autonomy (like other concepts like “democracy”, “justice” and “art”) is conceptualised differently by different authors.^1^ In the introduction, I suggested that autonomy was connected to rationality (Taylor 2009, 3; Savulescu 2007; Savulescu and Momeyer 1997), responsiveness to reasons, independence (Dworkin 1988), authenticity (Hyun 2001; Noggle 2005; Watson 1975), and the absence of coercion, deception and manipulation. Some authors argue that autonomy is inherently historical (Christman 1991) while others who hold structural accounts deny this, making autonomy depend on whether an individual’s preferences are “integrated” (Ekstrom 2005) or whether they are hierarchically ordered in the appropriate way (Dworkin 1988).

Anyone who delves into the philosophical literature surrounding autonomy quickly realises that there is no univocal understanding of autonomy to be found (Vargas 2006). Rather, “we find people disagreeing about the proper use of the concepts […] when we examine the different uses of these terms and the characteristic arguments in which they figure we soon see that there is no one clearly definable general use of any of them which can be set up as the correct or standard use” (Gallie 1956, 168). This, however, should not be taken to imply that discussions concerning autonomy are being conducted at cross purposes. Discussions of autonomy have, along with the lack of a single unified and standard use, another feature which distinguishes these forms of disagreement from disagreements over concepts which are radically confused. Even though different theorists have different competing interpretations of autonomy “a certain piece of evidence or argument put forward by one side in an apparently endless dispute can be recognized to have a definite logical force, even by those whom it entirely fails to convince” (Gallie 1956, 190).

In this intellectual climate, different accounts of autonomy, although they all attempt to capture some of the connotations that autonomy is taken to have, invariably end up being partially stipulative (Taylor 2009, 1–2; Double 1992), capturing most, but not all, of the elements of autonomy. In as far as these accounts fail to capture elements which are, for other theorists, central to autonomy, disputes arise not only about which conception of autonomy is better on its own terms, but also disputes about what the criteria of the “best account of autonomy” should be. Even though some of these revisionary accounts of autonomy can be quite successful they will inevitably fail to capture some of the elements commonly associated with autonomy. When discussing autonomy with other theorists who do not share the same set of intuitions and presuppositions about what autonomy is and why it matters, disagreements do not only centre on whether a particular account captures most of the connotations of the concept of autonomy but also whether these connotations are worth capturing (Gallie 1956, 171). Deeper disagreements appear when the term “autonomy” isn’t taken to be synonymous with “personal autonomy”. Once Kantian interpretations are incorporated, the possibility of extensionally correct elucidations of the term appears even more remote.

Although some of the elements that competing interpretations of autonomy attempt to capture are relevant to debates in bioethics and political philosophy, many often fail to adequately describe and justify the role autonomy currently plays in bioethical debate (Arpaly 2005). In as far as autonomy requires integrated preferences, independence and authenticity, these conceptions of autonomy ascribe autonomy to a relatively small subset of people. Importantly, this subset of people will be smaller than the group of people we (pre-theoretically) believe should be entitled to make their own decisions regarding their own healthcare in light of their own values and life plans. The problem with having divergent and mutually incompatible accounts of autonomy, hence, is that as a result of the theoretical discussions about which conception of autonomy is preferable they end up imposing requirements which conflict with what I take to be the primary role of autonomy within a liberal bioethics: the protection of people’s ability to live their own lives in accordance with their own values. As these competing interpretations are developed partly in response to theoretical debates which go beyond the protection of an individual’s ability to live a life plan and are, hence, much richer concepts; their inability to correctly identify the set of people who should be entitled to make their own decisions is to be expected.

Having outlined the structure of the problem, it is now time to turn to justifying its major premise: the role of autonomy in bioethics is protecting people’s ability to live their own lives in accordance with their own values. Increased usage of the term “autonomy” over the last 50 years within bioethics is partly a response to increased pluralism. Unlike in more homogenous societies, the citizens of pluralistic societies hold many diverse views about the good life which can, on occasion, violently conflict with the conception of the good held by other citizens of their polity. The term “autonomy” has come to play an anti-paternalistic role within bioethics as its value was appealed to as a means of justifying allocating decision-making authority over medical interventions to the person undergoing them. In virtue of the function autonomy has played in bioethical debates over the last half a century, usage within bioethics has distanced itself from the wider debates surrounding the meaning and value of autonomy, focusing in on the problem of who should decide what should happen to people in clinical contexts.

Increased pluralism, hence, has led to talk of autonomy being primarily about allocating decision-making authority to allow individuals with values different to the mainstream to live their lives in light of them. Debates about who should get to have decision-making authority over their healthcare in clinical bioethics do not focus on ascertaining whether or not individuals were brainwashed early in life, how their wills are structured or whether their autonomy is undermined in virtue of them having internalised beliefs which preclude them from choosing certain ends. Respect for autonomy in current liberal bioethics requires merely that we allow “adult, competent patients to base their decisions on whatever values they choose to structure their lives” (May 2005, 302) and that we “recognize with due appreciation that person’s capacities and perspective, include the right to control his or her affairs, to make certain choices, and to take certain actions based on personal values or beliefs” (Beauchamp 2005, 311). In conclusion, in a liberal bioethics, autonomy plays an important anti-paternalistic role by demarcating the actions “a patient has the right to perform without paternalistic intervention, actions that are ‘one’s own business’” (Arpaly 2005, 174).

In this section, I have argued that the primary function talk of autonomy plays in liberal bioethics (i.e., the protection of people’s abilities to live out their own lives in accordance with their own values) cannot be adequately explained and justified by the multiple, competing accounts of autonomy we find in the wider philosophical literature. This is due to the fact these conceptions of autonomy attribute the property to fewer people than those we (pre-theoretically) believe should be entitled to make their own decisions regarding healthcare. This lack of extensional correctness, I suggested, is due to the fact that conceptions of autonomy are constructed as a response to theoretical disputes which extend beyond the questions concerning who should be entitled to make their own decisions in pluralistic societies.

## Competence and Decisional Authority

Having argued that talk of autonomy fails to capture what I take to be the primary role of autonomy, it is time to turn to the second part of the argument: that competence is better suited to play the anti-paternalistic role currently assigned to autonomy. This argument will occur over the next two sections. In this section, I will argue that competence determinations which distribute decisional authority can protect an individual’s ability to live their own lives in accordance with their own values by grounding rights to non-interference. These rights to non-interference protect an individual’s ability to pursue their own conception of the good by providing them with a protected sphere of action in which they can pursue their projects without these being thwarted by others. Having shown that competence determinations which distribute decisional authority have to distribute rights to non-interference to protect people’s ability to live their own lives, in the section entitled “Risk-Sensitive Competence”, I take up the question of how we should assess competence. This second question is vital for the plausibility of the account being presented as depending on how competence is assessed, different sets of people will possess rights to non-interference. In light of the importance of these rights, the plausibility of substituting talk of autonomy with an account of competence depends heavily on who is deemed to be entitled to rights to non-interference. Having outlined how the positive argument is divided over the coming two sections, let us turn to arguing that the distribution of decisional authority protects an individual’s ability to live their own lives in accordance with their own values by grounding rights to non-interference.

The literature on competence widely agrees that the purpose of competence assessments is to ascertain who should have decisional authority over a particular choice (Kim 2006, 93; Richardson 2010, 62; Welie 2001, 139; Baumgarten 1980, 180; Buller 2001, 93; Annas and Densberger 1984, 561; Brock 1991; Skene 1991). Faden et al., for example, state that a judgement that one is competent “commonly functions to denote persons whose consents, refusals and statements of preference will be accepted as binding” (1986, 290). It is the purpose of this section to argue that possessing decisional authority is important as it enables those who possess it to live their own lives in accordance with their own values by making the person’s consent necessary before proceeding to act in certain ways. In as far as it is impermissible to go against the wishes and desires of competent people, a demonstration of competence entitles individuals to pursue their own plans in accordance with their own values by giving them power over what will happen to them. In other words, they grant individuals rights to non-interference.

In order to protect a person’s ability to live their own lives in accordance with their own values we must abstain from interfering with the choices individuals make as these are the means through which a person’s life plans are put into action. This is due to the fact that, in their absence, attempts to put complex plans into action could be thwarted by others (e.g., doctors, family members…) thereby reducing the extent to which people can live their lives in accordance with their own values. In order to give some definite content to the idea that the ability to live one’s life in accordance with one’s own values requires rights to non-interference, it is first necessary to see what rights to non-interference preclude others from doing (i.e., we need to know the scope of the right to non-interference). Establishing the scope of rights to non-interference is partly a tightrope act. If the rights are too robust, they will preclude interference with people’s choices in cases where we believe it permissible to interfere. If, on the other hand, the rights are too weak, the individual’s ability to live their own life will be too restricted. Keeping these two potential pitfalls in mind, it is time to answer the question: What constitutes interference?

In this paper, I will consider three types of interference with a person’s actions: Checking Interference (CI), Temporary Blocking Interference (TBI) and Permanent Blocking Interference (PBI). Let us call CI that aimed at ascertaining whether the individual is competent and Blocking Interference that aimed at precluding the individual from carrying out their proposed plan of action. Blocking Interference can be both Temporary (TBI) or Permanent (PBI). PBI occurs when an individual blocks another individual from pursuing their proposed plan of action indefinitely. TBI involves the delaying of the action for a definite and finite period of time. With this terminology in hand, let us consider a series of cases in order to see which forms of interference our rights to non-interference should protects us from.

- *Voluntary Amputation* Barry contacts his local surgeon to schedule a voluntary amputation of his leg. When the surgeon meets Barry, she tells him they won’t be performing the operation that day (TBI) as she first needs to establish that Barry is competent (CI). Barry, following a series of interviews with different medical practitioners, is deemed incompetent by the clinical team. In virtue of his incompetence, the clinical team make a recommendation on his medical history that he not be allowed to seek voluntary amputation from any other surgeon (PBI).
- *Wannabe* Maude has long wanted a surgical amputation of her leg and, hence, arranges an appointment with a surgeon. When the surgeon meets Maude, she tells her they won’t be performing the operation that day (TBI) as she first needs to establish that Maude is competent (CI). Maude is subjected to a series of interviews with different medical practitioners in which she explains why she believes she will be more comfortable having only one leg, why she believes surgery is for her, and that she is aware of both the potential negative health consequences and the likely inconveniences that she would inflict upon herself. Following these interviews, Maude is deemed competent by her clinical team. In virtue of her competence, the clinical team go ahead and arrange for a safe amputation.^2^

In *Voluntary Amputation* and *Wannabe*, both Maude and Barry are subjected to CI and TBI. TBI is often necessary in order for medical practitioners (and other bystanders in non-clinical contexts) to have the time to undertake CI. The difference between the cases was that Maude, following CI, was allowed to pursue her preferred course of action whereas Barry was not. This is because, in the examples, Barry is not competent, whereas Maude is. This is, I believe, intuitively correct. Whereas it is permissible to preclude Barry from amputating his leg, it is not permissible to preclude competent patients like Maude from doing so. This intuition lends support to the idea that a determination of competence should distribute decisional authority. Maude, as she is competent, should have the decisional authority to pursue her preferred course of action. Engaging in PBI with regards to his choice to seek a voluntary amputation would be impermissible as it would undermine his decisional authority. Maude should, hence, have a Right to Permanent Blocking Non-Interference (RPBI). Barry, in virtue of his incompetence, does not have a right against PBI. The RPBI, however, is not enough to adequately protect an individual’s decision-making authority. If Maude only acquired a right against PBI, it would be permissible to continue to engage in CI and TBI every time she wished to pursue courses of action which she has demonstrated her competence to perform. This is no small problem. Continuing to delay Maude’s plan of action through CI and TBI would interfere massively with her ability to live her own life in accordance with her own values. CI and TBI are instances of interference we should be protected from because we care *when* a project gets completed, not just *that* it does. In as far as our life plans involve coordinating action across time, forms of interference which delay the completion of parts of our plan interfere with our ability to live our own lives. If the role of competence is to protect our ability to live our own lives, the demonstration of competence should protect us from all three forms of interference: CI, TBI and PBI. In other words, the demonstration of competence grounds a Complete Right to Non-Interference (RCI + RTBI + RPBI). This right is a cluster right (Thomson 1990, 55) composed of three separate rights: the right to checking non-interference (RCI) and the rights to both temporary (RTBI) and permanent blocking non-interference (RPBI).

When an individual possesses a Complete Right to Non-Interference (RCI + RTBI + RPBI), they possess decisional authority over their choices, which makes them “persons whose consents, refusals and statements of preference will be accepted as binding” (Faden et al. 1986, 290). The demonstration of competence, by grounding rights to non-interference, grants individuals a sphere in which to pursue their own lives in accordance with their own values and, hence, is able to play the anti-paternalistic role commonly attributed to autonomy. Having argued that the demonstration of competence grounds Complete Rights to Non-Interference (RCI + RTBI + RPBI) as a means to protecting an individual’s ability to live their own lives, it is time to turn to answering the most important question: How do individuals demonstrate competence? This question is crucial as the answer to it determines which individuals are entitled to Complete Rights to Non-Interference (RCI + RTBI + RPBI) and, by extension, who is protected in the pursuit of their own lives in accordance with their own values.

## Risk-Sensitive Competence

In the preceding section, I argued that in order for competence determinations to distribute decisional authority in such a way as to protect an individual’s ability to live their own lives in accordance with their own values, they must ground Complete Rights to Non-Interference (RCI + RTBI + RPBI). In virtue of the role competence assessments play, whether or not an individual is deemed competent has substantial normative implications. As was seen in *Voluntary Amputation*, failing to demonstrate one’s competence can lead to PBI and more CI which, I argued above, substantially impair an individual’s ability to live their own lives. In virtue of the contribution a demonstration of competence makes to people’s ability to pursue their own life plans, it is vital to provide an account of how an individual demonstrates competence in order to see whether or not the account of competence presented can take on autonomy’s anti-paternalistic role. If the account of competence fails to distribute decisional authority to all those who we (pre-theoretically) believe should be entitled to make their own healthcare decisions, it will fail as a substitute for talk of autonomy which, in virtue of its wider theoretical baggage, leads to extensionally incorrect distributions of decisional authority.

In this section, I argue for a risk-sensitive account of competence as a criteria for distributing decisional authority. On a risk sensitive account of competence an individual demonstrates their competence by exercising their agential capacities to the extent necessary to meet a threshold determined by the riskiness of the decision. The three capacities the individual must exercise are: (i) the capacity to acquire knowledge, (ii) the capacity for instrumental rationality, and (iii) the capacity to form and revise a life plan.

Before turning to specifying how risk influences the threshold of capacity an individual has to meet to demonstrate competence I will, firstly, explain why the exercise of capacities (i) through (iii) is necessary and sufficient for an individual to be considered competent to consent.

### Knowledge, Rationality and a Life Plan

In order for individuals to be competent to pursue a course of action, they must possess the ability to acquire and understand factual knowledge about the world. This, in turn, will require: the capacity to perceive the world in a way which allows for new evidence to become salient (Savulescu and Momeyer 1997), memory, the capacity to represent the world in the form of beliefs and, finally, the ability to contrast beliefs with reality to ascertain whether they are true.

The capacity to perceive the world in some way is a sensible component of the capacity to acquire knowledge as, without it, the only knowledge we would have access to would be either a priori, or the result of introspection. In order for an individual to be deemed competent to make the types of choices involved in clinical care they require knowledge of the world, not just a priori knowledge. Memory is also important for competence in virtue of the fact that medical decisions extend over time. Without the capacity to retain the information required, we would be unable to put our life plans into effect. With regard to the capacity to form beliefs and contrast them to the world, these are required due to the fact knowledge consists of, at least, justified true belief (Gettier 1963). Without the capacity to represent the sense data we receive and the inferences we draw from it as beliefs, we would be unable to meet one of the necessary conditions of knowledge: the possession of beliefs about the world. In order to acquire knowledge, we must also possess the ability to contrast these beliefs with reality in order to be justified in believing them (using our sensory capacities, memory and reason). In as far as justification has something to do with a relationship between the belief and the world, the ability to contrast beliefs to the world is necessary for the capacity to acquire knowledge. Exactly which mental faculties are involved in producing reliable methods of contrasting beliefs to the world (i.e., the question of when we are justified in believing propositions) is a question which goes beyond the concern of this paper. The reader is, hence, invited to use their favoured theory of justification to answer these questions. Whilst the capacity to acquire factual knowledge seems necessary for an individual to be deemed competent, it is not sufficient. Without the capacities for instrumental rationality and the capacity to form and revise a life plan, we lack a set of ends to pursue and the ability to choose means in light of them. Unless an individual can demonstrate the exercise of these two further capacities, they cannot acquire Complete Rights to Non-Interference (RCI + RTBI + RPBI).

Let us now turn to the second capacity, the capacity for instrumental rationality. This capacity, like the capacity to acquire knowledge, is necessary for an individual to be deemed competent. Without the ability to use the information acquired in decision-making to pursue one’s ends, the capacity for knowledge does not give us the means necessary for us to be able to live our own lives in accordance with our own values. Instrumental rationality involves the ability to compare the representations we make of courses of action in light of our ends (Berg et al. 1996; Appelbaum 2007). The capacity for instrumental rationality, hence, requires the ability to join factual information with questions of value in order to arrive at conclusions about what we have reason to do. As the demonstration of competence grounds Complete Rights to Non-Interference (RCI + RTBI + RPBI) as a means of providing us with the space to pursue our life plans, in order to be deemed competent an individual must be able to exercise the capacity to reason instrumentally. If the capacity for instrumental rationality is not taken as an element of competence, there would not be a connection between the rights demonstrating competence grounds and the ability to pursue one’s life plan that the rights protect. The exercise of the capacity for instrumental rationality is, along with the capacity to acquire factual knowledge, necessary for an individual to be deemed competent and, hence, acquire Complete Rights to Non-Interference (RCI + RTBI + RPBI). These two capacities, whilst both necessary, are not jointly sufficient.

The third capacity which constitutes competence is the capacity to form and revise a life plan. Without a life plan to provide the ends, instrumental rationality and the capacity to acquire knowledge would be unable to provide guidance regarding which course of action to take. Without a life plan, people would be unable to evaluate states of the world and (using their instrumental rationality and knowledge) choose in light of these evaluations. By organising our diverse values into more of less coherent wholes (Fried 1970, 19), life plans provide us with the evaluative framework necessary to rank alternative courses of action, which is a precondition to choosing in such a way as to pursue our conceptions of the good. The values life plans organise are diverse in a number of ways. Values vary in their generality (Raz 1986, 293) and in their fundamentalness (Noggle 1997, 318). General values permeate more aspects of our lives than their more specific counterparts. Honesty, for example, could (and arguably should) influence our decisions in all areas of our life, whilst chastity may only affect our sex lives (and/or religious lives if our particular religion proscribes certain sexual practices, such as sex outside marriage). Fundamental values come closer to the core of who we are than superficial values and are those we are less inclined to give up. Further along the scale of fundamental-ness are “grounding projects”. Our “grounding projects” are those values which are so fundamental there may be no reason to carry on living in their absence (Williams 1976, 209).

The diversity of values requires a system for ranking these and determining “the magnitudes of risk which he will accept for his various ends at various times in his life” (Fried 1970, 95; Childress 1982, 191). Life plans, hence, govern the trade-offs between goods which cannot be pursued in parallel (Dworkin 2006, 109). When faced with a choice which requires a trade-off, having an organised system of values enables us to consider how factual information that informs choices is relevant to our lives by asking, for example, whether a particular sacrifice of one value is warranted in virtue of its contribution to furthering another and whether the choice violates one’s “ground projects”.

The principles which rank alternatives and stipulate how much risk to one goal we are willing to accept for another are often hard to express propositionally (Raz 1986, 294) and, for that reason, often remain implicit or inchoate (Fried 1970, 30). In as far as these principles remain inchoate they are vulnerable to two types of problem: incompleteness and inconsistency. Situations may present themselves which call for a ranking of values or states of affairs which we have never considered or even for the valuing of something which we had not considered valuing before. These tensions provide us with opportunities to extend our partially inchoate life plan, making it more comprehensive. Novel situations can also provide evidence of inconsistency in one’s life plan. When our partially complete ordering of values and ends appears to recommend two incompatible courses of action, we need to modify our life plans to reduce this inconsistency. These two problems generated by the inchoate nature of our life plans make the capacity to revise, as well as to form, life plans crucial to whether an individual is competent.

Competence, hence, is determined by the extent to which an individual exercises three capacities: (i) the capacity to acquire knowledge, (ii) the capacity for instrumental rationality, and (iii) the capacity to form and revise a life plan. These three capacities, I have argued, are individually necessary and jointly sufficient for a person to be competent. Whether or not an individual is competent, I stated above, was a matter of whether or not the individual could meet a threshold. The use of thresholds is necessary for scalar properties to yield binary determinations. As the exercise of the three capacities which constitute competence is a matter of degree; in order for a demonstration of competence to distribute decisional authority in a binary fashion, we need to set a threshold which, once met, would entitle the individual to Complete Rights to Non-Interference (RCI + RTBI + RPBI). In virtue of the significance of meeting the threshold that grounds RCI + RTBI + RPBI, the open question at this point is: How do we set the threshold?

### Risk and Thresholds

In this section, I will argue for a risk-sensitive approach to competence (Cale 1999; Brock 1991; Skene 1991) as a means of setting the threshold of the three capacities which constitute competence which the individual must meet to ground their Complete Rights to Non-Interference (RCI + RTBI + RPBI). The basic claim of a risk-sensitive approach to competence is the following: the riskier the decision, the greater the extent to which an individual must demonstrate the exercise of the capacities which jointly constitute competence in order to acquire a Complete Right to Non-Interference (RCI + RTBI + RPBI). On this account, it is easier to demonstrate competence for non-risky courses of action than it is for risky ones. As the demonstration of competence determines whether we possess a Complete Right to Non-Interference, it will be harder to pursue risky courses of action without being interfered with than it will be to pursue non-risky ones. In light of the vital role Complete Rights to Non-Interference play in enabling us to pursue our plans, any measure which makes it harder for people to acquire them stands in need of justification. The question of how the riskiness of a decision is determined is, hence, central to the plausibility of the account presented. If we determine risk in the wrong way, we may end up denying Complete Rights to Non-Interference to individuals who should, intuitively, be entitled to live their own lives in accordance with their own values.

In this section, I will argue that the extent to which a proposed activity is risky for an individual should be determined using the individual’s own values (Buchanan and Brock 1986). On a subjective account of risk, the riskiness of a decision is determined by assessing the probability of an outcome happening versus the magnitude of the harm. The magnitude of the harm is to be determined by the extent to which the outcome would negatively impact a person’s life plans and their ability to pursue them. This is, I believe, required if competence is to play the anti-paternalist role currently attributed to autonomy. The role of autonomy in a liberal bioethics, I argued above, is to protect an individual’s ability to live their own lives in accordance with their own values. As individual’s life plans contain ranking principles which mark out some goods as being more important than others (Fried 1970, 19; Raz 1986, 292) and set the magnitude of risk to one goal one would be willing to take in order to complete another (Fried 1970, 95, 177; Childress 1982, 191), respecting an individual’s ability to pursue their own life requires setting the threshold of competence according to the individual’s own values. If, instead of using a subjective approach to risk, we used an objective determination of risk (medical best interests, for example), we would fail to respect an individual’s ability to live their life plans in accordance with their own values. As the riskiness of the decision sets the threshold the individual must meet to acquire Complete Rights to Non-Interference (RCI + RTBI + RPBI), using an objective criterion of risk could lead to situations in which individuals are found to be incompetent (with its corresponding loss of decision-making authority) on the basis of the threshold being raised in response to a factor the individual does not consider to be risky. This, I believe, fails to respect an individual’s ability to live their lives in accordance with *their* values.

With a sketch of the theory in hand, let us return to some modified versions of the cases involving voluntary amputation presented above to see whether the account presented here is plausible.

- *Magician* A magician consults his surgeon and asks for them to amputate his hand, stating he no longer wants it to be a part of him. Presented with such an unusual request, the surgeon refers the patient to a psychiatrist who interviews the magician to determine whether he is competent (CI + TBI). In order to do so, the psychiatrist first inquires into the magician’s values and plans. The psychiatrist discovers the magician often does card tricks during his performances and, although currently pursuing an alternative career as a barista, still has dreams of becoming a full time magician. During the course of the interview, it becomes apparent the magician is uncomfortable accepting help from others, even with tasks he struggles with, such as filling out his taxes. This behaviour coheres with his expressed desires to be independent and not the object of charity. It is deemed by the clinical team that, in light of a hand amputation being hard to make compatible with the magician’s values and desires, the procedure is risky for the magician. A suitably high threshold of competence is then established. Having decided to go ahead anyway, the magician’s competence is assessed. Upon failing to meet the high threshold, the individual is deemed incompetent and the operation fails to go ahead.
- *Leg Amputation* Sandra has long wanted a surgical amputation of her leg and, hence, arranges an appointment with a surgeon. Presented with such an unusual request, the surgeon refers the patient to a psychiatrist who interviews Sandra to determine whether she is competent (CI + TBI). In order to do so, the psychiatrist first inquires into Sandra’s values and discovers she has long been a *wannabe* (i.e., an individual who desires a voluntary amputation). Furthermore, she has experimented for periods of time with restricting the movement of her leg, using crutches and wheelchairs to move around the house. Although the pursuit of other activities she values is made more cumbersome with the walking aids, she is willing to pay these costs for satisfying her persistent desire for a voluntary below the knee amputation if a prosthesis could not be fitted. Following this interview, the voluntary amputation of Sandra’s leg is not deemed particularly risky to Sandra and a suitably low threshold (which the magician could have met) is established. Deciding to go ahead with the amputation, Sandra’s competence is assessed, she is deemed competent and the surgeon proceeds with the amputation.

An important implication of the account being presented here is the fact that a risk-sensitive account of competence entitles people to pursue actions which are compatible with their life plans relatively free from interference. Actions which are not compatible with the individual’s life plan are, on a risk-sensitive account, correspondingly hard to pursue. In *Magician*, the protagonist was deemed incompetent to pursue his plan in virtue of the fact that the procedure was risky for him. Whereas in *Leg Amputation,* Sandra was entitled to pursue her proposed plan of action by meeting a low threshold, the magician was precluded from engaging in qualitatively similar actions on the basis of his inability to meet a high threshold. It seems, then, that the magician’s failure to acquire Complete Rights to Non-Interference is *caused* by the fact competence has been taken to be risk-sensitive. It could be objected that, under an alternative account of competence which didn’t make reference to the riskiness of the decision, the magician would have been entitled to pursue his amputation free from interference by others. This objection can be interpreted as a demand for justification for raising the threshold. Why, then, is it the case that Sandra should have decision-making authority to pursue her voluntary amputation whereas the magician should not?

The reason Sandra should be entitled to have a voluntary amputation is due to the fact that this option is an important element of her life plan. Not allowing the individual to pursue these choices would unjustifiably limit her ability to live her life in accordance with her own values. The importance of allowing individuals to live their own lives does not disappear when the choices they make appear to be harmful or strange. Complete Rights to Non-Interference (RCI + RTBI + RPBI) protect an individual’s ability to live their lives regardless of what others perceive to be the “correct” choice. The right to non-interference grants the individual a sphere of jurisdiction in which “we let that person go his own way, whether we approve of it or not” (Neumman 2000, 294). This, I believe, is in keeping with the role autonomy should play in a liberal bioethics. The respect we have for autonomy in clinical encounters is not a form of appraisal respect, but of recognition respect. Appraisal respect, unlike recognition respect, is a response to excellence (Hill 2000) and, hence, requires pro-attitudes towards the object of respect. Recognition respect, on the other hand, is a matter of giving the object of respect appropriate weight in one’s deliberations and acting accordingly (Darwall 1977; Dillon 2016). In order to give people’s capacities appropriate weight, we must not preclude them from pursuing their projects. Importantly, this requirement holds independently of what we think of the content of the individual’s life plan. If competence is to take the role of autonomy in a liberal bioethics, it too must ground rights independently of whether or not others approve of the life plan they will be used to pursue. Having shown that Sandra should be entitled to pursue her life plan, let us now turn to see why it is appropriate to not give Complete Rights to Non-Interference (RCI + RTBI + RPBI) to the magician.

Not giving complete rights to non-interference to the magician is justified in virtue of the fact that raising the threshold of competence in the way required by the risk-sensitive standard does not preclude the magician from living out his own life in accordance with his own values. In as far as the threshold has been raised using the individual’s own values, not granting the individual Complete Rights to Non-Interference (RCI + RTBI + RPBI) if he decides to act against his expressed values is, if anything, more akin to *requiring* him to live in accordance with his values rather than prohibiting him from living his life in accordance with them. Raising the threshold in this way, hence, is compatible with the role autonomy should play in a liberal bio-ethics, i.e., protecting people’s abilities to live their own lives in accordance with their own values.

It could be objected, however, that requiring people live their lives in accordance with their own values is also problematic in virtue of the fact it limits the extent to which an individual is free to choose which course of action to pursue. In light of the common association between respect for autonomy and allowing individuals to choose which course of action they want to take (Taylor 2009, 83), requiring an individual live their own life in accordance with their own values could be interpreted as incompatible with respect for autonomy and an infringement of the “freedom of each person to order her life and constitute her self in her own way” (Noggle 1997, 509).

This interpretation, however, should be resisted. Although respect for autonomy normally requires a broad range of choices, it is not the same as merely providing negative space in which people can act (Taylor 2009, 21). Autonomy requires more than that. If autonomy and negative liberty could be equated, paradigmatically non-autonomous agents (e.g., wantons)^3^ could be deemed to be autonomous, as nothing precludes them from possessing negative liberty.

Not being able to equate negative liberty and autonomy, it is no longer obvious that raising the threshold of competence in light of the individual’s values is incompatible with respect for autonomy. In as far as autonomy includes something more than mere negative liberty, it is not unreasonable to suppose that protecting an individual’s ability to live one’s life in accordance with their own values could require not allowing people to act against their expressed values without demonstrating the exercise of the capacities to (i) acquire knowledge, (ii) reason instrumentally, and (iii) form and revise a life plan to the extent necessary to meet a high threshold. This is due to the fact that, “given a person’s particular motivational set”, an individual may be better able to exercise their autonomy were they “to have a set of options available to her that had a smaller number of elements than some alternative-sets” (Taylor 2009, 94). A risk sensitive threshold of competence, insofar as it uses the individuals own values, is compatible with respect for autonomy even if it does, in cases like *Magician,* preclude certain individuals from pursuing courses of action which run counter to their expressed values. This is due to the fact that the reason why the threshold of competence is raised is particular to each individual and, hence, is a response to *their* ability to live their lives in accordance with their own values.

In the section entitled “Knowledge, Rationality and a Life Plan”, I argued that competence is demonstrated by meeting a threshold of the three capacities: (i) the capacity to acquire factual knowledge, (ii) the capacity for instrumental rationality, and (iii) the capacity to form and revise a life plan. In “Risk and Thresholds”, I argued that protecting an individual’s ability to live their own lives in accordance with their own values required the use of a subjective criterion of risk, where the riskiness of the decision is determined by the extent to which the proposed course of action interacts negatively with the individual’s own values. With a sketch of the account in hand, I turned to discussing two cases and argued that, even when the risk-sensitive account of competence raises the threshold an individual has to meet in such a way as to preclude them from carrying out a particular action, this is in line with what is required by respect for autonomy as respect for autonomy is compatible with limiting the option sets of particular people.

## Conclusion

In this paper, I have argued for a risk-sensitive account of competence as a substitute for “autonomy” in clinical ethics. This account of competence has been shown to protect people’s ability to live their own lives in accordance with their own values by grounding Complete Rights to Non-Interference (RCI + RTBI + RPBI). As the protection of people’s ability to live their own lives is the primary role of autonomy in a liberal bioethics, the account of competence argued for here is capable of doing the work currently attributed to autonomy (i.e., distributing decisional authority to particular people over particular choices as a means of protecting their ability to live their lives in accordance with their own values).

Moreover, in light of the fact that different conceptions of autonomy attribute the property to different sets of people in ways that conflict with our (pre-theoretical) beliefs about who should be entitled to make their own decisions regarding their own health care; using an account of competence to draw the limits of permissible paternalism allows us to do so without being influenced by the wider theoretical debates accounts of autonomy are developed in response to. Substituting autonomy for competence allows us to focus more closely on the question of who should be entitled to make their own healthcare choices, without being influenced by discussions about how coherent, integrated or authentic an individual’s preferences are; all of which are orthogonal to the issue of who should be given decisional authority.

## Footnotes

1 Authors such as Vargas (2006), Arpaly (2003), Dworkin (1988) and Rainer Forst (2005) propose extensive taxonomies of different types of autonomy as a means of illustrating how varied the conceptions of autonomy are. These extensive taxonomies of different uses, however, are disputed by other authors. Taylor (2009), for example, has argued that Arpaly’s (2003) taxonomy in particular is based on a systematic misunderstanding of the nature of autonomy.

2 This case is not dissimilar from the case of Robert Smith, who performed a healthy limb amputation for a patient in 1997 (Bayne and Levy 2005, 75). For further discussion of the ethics of voluntary amputation see: Patrone (2009), Bridy (2004), Song (2013), Ryan (2009), Schramme (2008), White (2014).

3 A “wanton” is a type of being which, although it has first order desires (e.g., “I want a cake”), does not possess any second order desires (i.e., desires about desires) about which of its first order desires should be its will. A wanton, when confronted with two first order desires which conflict (such as “I want cake” and “I don’t want to put on weight”) has no desires about which of these two desires should become its will.
